# The winners project: neuropsycological changes after a video game-based training program in pediatric cancer survivors. a case report

**DOI:** 10.1192/j.eurpsy.2024.1345

**Published:** 2024-08-27

**Authors:** C. Gonzalez-Perez, E. Moran, N. Malpica, J. Alvarez-Linera, H. Melero, M. Alonso, M. Esteban, A. Perez-Martinez, E. Fernández-Jiménez

**Affiliations:** 1 Department of Pediatric Hemato-Oncology, La Paz University Hospital; 2 Children Psychology, Juegaterapia; 3 Medical Image Analysis and Biometrics Laboratory (LAIMBIO), Rey Juan Carlos University; 4 Department of Neuroradiology, Hospital Ruber Internacional; 5 Psychobiology and Methodology in Behavioral Sciences, Complutense University; 6 Juegaterapia; 7 Department of Psychiatry, Clinical Psychology and Mental Health, La Paz University Hospital, Madrid, Spain

## Abstract

**Introduction:**

Children who have undergone an oncological process and have received treatment with chemotherapy or radiotherapy on the central nervous system may have significant neurocognitive sequelae. Some video games have shown neurocognitive benefits in people with impairments in different areas, such as attention or memory.

**Objectives:**

This work aims to demonstrate the benefit of a video game-based training program to improve the neurocognitive profile in a child survivor of cancer.

**Methods:**

The patient is a 9-year-old female who was diagnosed with acute lymphoblastic leukemia at the age of 4 years. She received routine treatment of this disease by chemotherapy, including high-dose chemotherapy (with blood-brain barrier crossing) and intrathecal chemotherapy. She is currently 3 years after the end of treatment.

The Continuous Performance Test 3 (CPT-3) (sustained attention/vigilance) was administered before and after a multifaceted training program consisting of playing 3 video games for 12 weeks, as follows: a brain-training game (4 days per week, 7-12 minutes per day), a skill-training game (2 days per week, 10 minutes per day) and an exergaming game (2 days per week, 10 minutes per day).

**Results:**

Prior to intervention, the patient had 3 atypical z-scores on the CPT-3 (z scores: mean = 0, S.D. = 1), with a pattern compatible with ADHD (omissions z = 1.2; hit reaction time z = 3.4; hit reaction time block change z = 1.2). After intervention, she had only an atypical z-score (hit reaction time z = 3.6), with a pattern compatible with slowing, without ADHD.

**Conclusions:**

The neuropsychological evaluation of this patient showed an improvement in his attentional pattern on the CPT-3 after the video game-based training.

**Disclosure of Interest:**

None Declared

